# Ferroptosis and noncoding RNAs: exploring mechanisms in lung cancer treatment

**DOI:** 10.3389/fcell.2025.1522873

**Published:** 2025-02-26

**Authors:** Nadi Rostami Ravari, Farzad Sadri, Mohammad Ali Mahdiabadi, Yaser Mohammadi, Zahra Ourang, Zohreh Rezaei

**Affiliations:** ^1^ Department of Animal Science Researches, Agriculture and Natural Resources Education and Research Center of Kerman, Agriculture and Natural Resources Education and Research Organization (AREEO), Kerman, Iran; ^2^ Student Research Committee, Birjand University of Medical Sciences, Birjand, Iran; ^3^ Geriatric Health Research Center, Birjand University of Medical Sciences, Birjand, Iran; ^4^ Department of Internal Medicine, School of Medicine, Birjand University of Medical Sciences, Birjand, Iran; ^5^ Department of Biochemistry, School of Medicine, Iran University of Medical Sciences, Tehran, Iran; ^6^ Department of Biochemistry, School of Medicine, Arak University of Medical Sciences, Arak, Iran; ^7^ Department of Biology, University of Sistan and Baluchestan, Zahedan, Iran

**Keywords:** ferroptosis, lung cancer, noncoding RNAs, therapeutic targets, metastasis

## Abstract

Lung cancer (LC) is a highly prevalent and deadly type of cancer characterized by intricate molecular pathways that drive tumor development, metastasis, and resistance to conventional treatments. Recently, ferroptosis, a controlled mechanism of cell death instigated by iron-dependent lipid peroxidation, has gained attention for its role in LC progression and treatment. Noncoding RNAs (ncRNAs), such as microRNAs (miRNAs) and long noncoding RNAs (lncRNAs), are emerging as key modulators of ferroptosis, significantly influencing LC biology. This review explores how ncRNAs control ferroptotic pathways and affect tumor growth, metastasis, and therapy resistance in LC. By understanding the dual functions of ncRNAs in both activating and inhibiting ferroptosis, we aim to uncover new therapeutic targets and strategies for LC. These insights provide a promising direction for the development of ncRNA-based treatments designed to induce ferroptosis, potentially improving therapeutic outcomes for patients with LC.

## 1 Introduction

Lung cancer is a common type of cancerous growth in humans, usually starting in the trachea, bronchus, or lungs. Lung cancer continues to be a predominant cause of death from cancer globally. In 2024, approximately 234,580 new cases are anticipated in the United States, resulting in around 125,070 fatalities (Kazerooni et al., 2024). Lung cancer is broadly classified into two primary types: small cell lung cancer (SCLC) and non-small cell lung cancer (NSCLC). The latter, constituting roughly 85% of lung cancer diagnoses, includes subtypes like large cell carcinoma (LCC), squamous cell carcinoma (LUSC), and lung adenocarcinoma (LUAD) (Yang and Fan, 2024). The precise causes of lung cancer are understood; however, they are commonly linked to elements such as lifestyle choices, environmental effects, genetic predispositions, and other related issues (Bade and Cruz, 2020). Early-stage lung cancer often lacks distinct pathological symptoms, presenting primarily respiratory issues like coughing, difficulty breathing, and chest pain, which can result in delayed diagnosis and treatment (Xing et al., 2023).

In recent years, researchers have placed significant emphasis on exploring the molecular processes that contribute to the advancement of cancer. Among these mechanisms, various forms of programmed cell death (PCD)—such as necroptosis, apoptosis, autophagy, pyroptosis, and ferroptosis—have been extensively studied. Ferroptosis has recently gained attention for its unique characteristics and potential role in cancer progression (Wei et al., 2023). Ferroptosis occurs as a result of lipid peroxidation mediated by divalent iron, ultimately causing cell death through the excessive accumulation of intracellular lipid peroxides. It is distinct in its mitochondrial and oxidative stress-related changes, including smaller mitochondria with dense membranes, elevated ROS, increased lipid peroxidation, and alterations in genes like GPX4 and TFR1 (Li et al., 2023; Tang et al., 2022; Zhang et al., 2024a). In contrast to normal cells, lung cancer cells demonstrate a greater need for iron to support their increased proliferation, and disruptions in iron metabolism can elevate cancer risk and facilitate tumor growth (Manz et al., 2016). Finding how the molecular dysregulation of ferroptosis contributes to tumor development, metastasis, and therapy resistance in lung cancer is critical for identifying novel therapeutic strategies. Consequently, inducing ferroptosis in lung cancer cells presents a promising approach to inhibit tumor growth and mitigate resistance to drugs and radiation therapy.

Non-coding RNA (ncRNA) transcripts may be able to control the ferroptotic process (Luo et al., 2021). NcRNA molecules are involved in iron metabolism and ferroptosis-related amino acid metabolism, both of which are crucial for regulating the ferroptotic flux (Fearnhead et al., 2017). Additionally, ncRNAs influence reactive oxygen species (ROS) metabolism, a key factor in ferroptosis, as the accumulation of intracellular lipid ROS is a significant trigger for this type of cell death (Badgley et al., 2020; Luo et al., 2021). Moderate increases in ROS levels can stimulate the growth and division of cells, their ability to survive, and their potential to become cancerous. While ncRNAs can control the levels of ROS in order to maintain a balanced redox state, a reduction in ROS can prevent a kind of cell death called ferroptosis (Luo et al., 2021). Thus, ncRNAs can either promote or suppress cancer progression by modulating ferroptosis, a critical process in the development of cancer (Hushmandi et al., 2024). This review will assess the regulatory mechanisms of ferroptosis, focusing on its influence on lung cancer under the influence of ncRNAs. It will explore how ncRNAs, including miRNAs, lncRNAs, and circRNAs, regulate ferroptosis by modulating iron, lipid peroxidation metabolism, and ROS levels. This analysis aims to highlight the potential therapeutic targets within these regulatory networks for lung cancer treatment.

## 2 Basic mechanism and targets in ferroptosis

The identification of ferroptosis originates from initial investigations into the functionality of the cystine/glutamate antiporter (system xCT), first recognized in 1980. This system enables cystine uptake in exchange for glutamate, serving a vital function in cellular antioxidant defense (Yan H.-f. et al., 2021). Early research showed that stopping the absorption of cystine could cause oxidative stress and start cellular apoptosis. This was first seen in neuronal cell lines that were exposed to glutamate. Subsequently, researchers discovered that antioxidant supplementation, including alpha-tocopherol (-toc), mitigates glutamate-induced damage (Jiang X. et al., 2021).

In 2003, a high-throughput small-molecule screening discovered erastin, a chemical that specifically causes apoptosis in RAS-mutant tumor cells (Dolma et al., 2003). However, the exact mechanism responsible for erastin-induced mortality remained unclear at that time. Subsequent research in 2008 discovered RAS-selective lethal molecules (RSL-3 and RSL-5) as agents capable of inducing cell death independently of conventional apoptotic pathways (Yang and Stockwell, 2008). Ferroptosis was officially designated as a distinct kind of cell death in 2012. Subsequent experiments demonstrated that erastin obstructs cystine uptake through system xCT, leading to lipid peroxidation within cells, driven by iron, and eventually causeing cell death (Murphy et al., 1989).

The important role of GPx4 in stopping ferroptosis by lowering lipid hydroperoxides was shown by researchers in 2014 (Yang et al., 2014). Researchers found that the iron-transporting protein transferrin and the metabolism of glutamine were very important for starting ferroptosis. On the other hand, stopping glutaminolysis or transferrin receptor function lowered ferroptosis and lessened the damage to the heart from ischemia/reperfusion (Zilka et al., 2017).

Recent research has emphasized how lipoxygenases (Lox) facilitate the oxidation of polyunsaturated fatty acids (PUFAs) and the critical involvement of PHKG2-dependent iron pools in this process (Ingold et al., 2018). In 2017, researchers recognized acyl-CoA synthetase long-chain family member 4 (ACSL4) as a biomarker and crucial factor in ferroptosis, as it regulates the production of PUFAs (Doll et al., 2017). Furthermore, the discovery of ferroptosis-suppressing protein 1 (FSP1) in 2018 revealed a way to stop ferroptosis that does not involve glutathione, which helped us understand the process even better (Doll et al., 2019).

**FIGURE 1:**
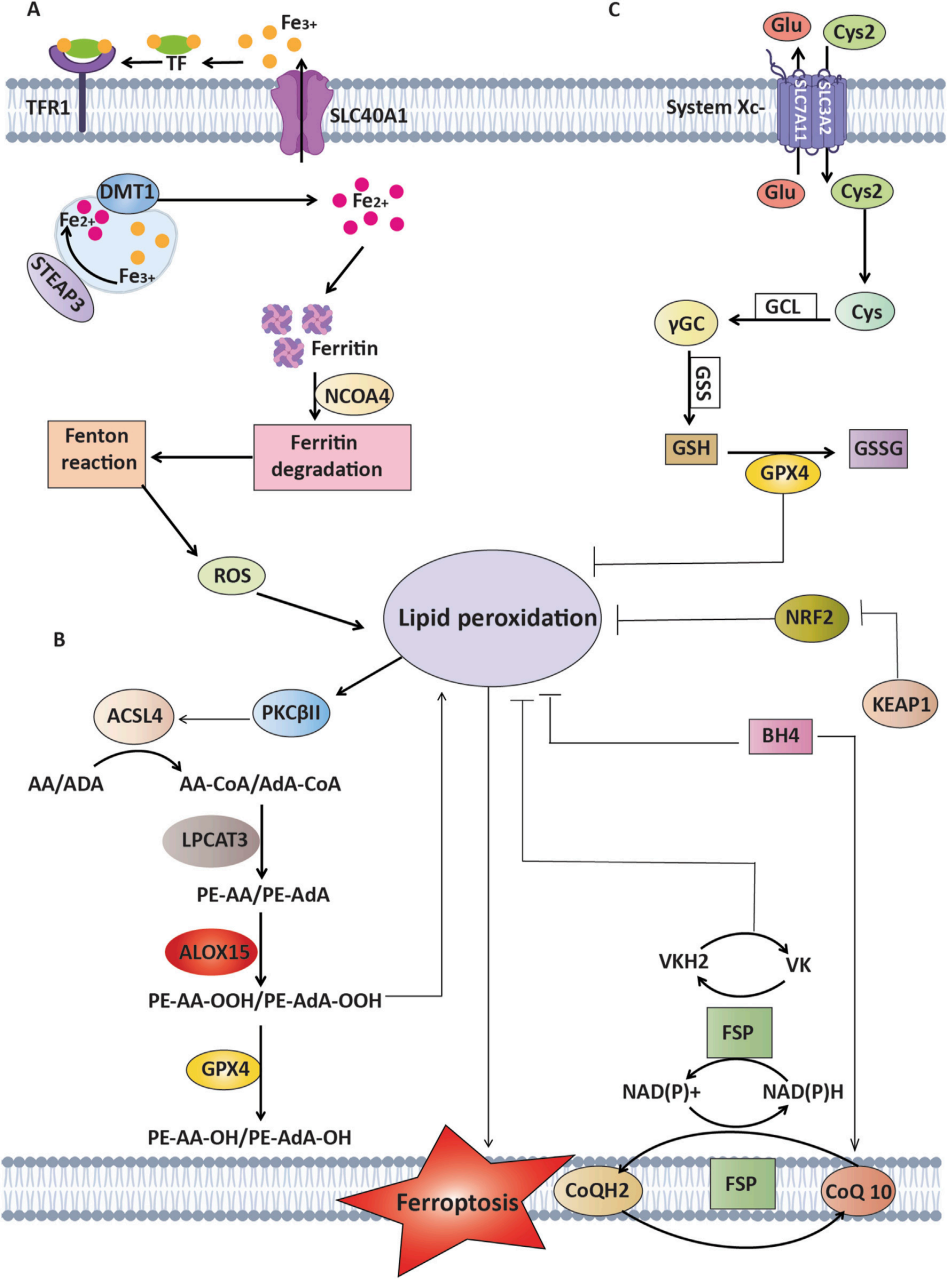
Mechanisms of ferroptosis and defense against lipid peroxidation pathways. This Figure represents the process of ferroptosis, which is driven by the oxidation of lipids that rely on polyunsaturated fatty acids (PUFAs) such as arachidonic acid (AA) and adrenic acid (AdA), in addition to ROS and iron. **(A)** Mechanisms of Iron Metabolism: Transferrin and its receptor (TFR1) play a crucial role in transporting Fe³⁺, whereas STEAP3 and DMT1 facilitate the transformation and transport of Fe^2^⁺, which ultimately results in lipid peroxidation. **(B)** Mechanisms of lipid peroxidation: Enzymes that consist of ACSL4 and LPCAT3 play a vital role in the formation of AA-PE/AdA-PE. PKCβII functions as a detector of lipid peroxidation and can increase lipid peroxidation through phosphorylating ACSL4. **(C)** Antioxidant Mechanisms: GPX4 enzymatically converts harmful lipid peroxides (PL-OOH) into harmless forms (PL-OH) using glutathione (GSH) as a helper molecule. This process is assisted by the cystine/glutamate antiporter xCT. FSP1 suppresses lipid peroxidation through the conversion of oxidized CoQ to CoQH2 without relying on GSH. Additionally, it activates a vitamin K cycle to combat ferroptosis. BH4 functions as an antioxidant without relying on GPX4 and FSP1. NRF2, a crucial regulator of antioxidant response, inhibits lipid peroxidation by increasing the expression of many ferroptosis inhibitors.

Researchers have recently found that peroxisomes make cells more vulnerable to ferroptosis by making polyunsaturated ether phospholipids (PUFA-ePL). These findings reveal the complex biochemical and molecular networks driving ferroptosis and their role in cancer biology (Zou et al., 2020a). They also emphasize the interconnected roles of iron metabolism, lipid peroxidation, and redox equilibrium in facilitating ferroptosis (Table 1) (Figure 1). The interaction between these pathways underlies this distinctive cell death mechanism, influencing its effects on cancer progression and treatment potential. Understanding how changes in these systems affect ferroptosis sensitivity is important for coming up with new ways to target cancer cells more specifically, especially those that are resistant to treatment.

**TABLE 1:** Key genes/proteins and processes in ferroptosis.

Gene/Protein	Target	Metabolomic process	Biological function	Cancer process	References
GPX4	Lipid peroxides	Detoxification of lipid ROS	Prevents lipid peroxidation and ferroptosis	Inhibits tumor growth	Imai et al. (2017), Stockwell et al. (2017)
SLC7A11 (xCT)	Cystine uptake	Glutathione synthesis	Maintains redox balance	Enhances drug resistance	Koppula et al. (2021), Chen et al. (2020)
ACSL4	PUFA-CoA synthesis	Lipid metabolism	Promotes lipid peroxidation	Increases ferroptosis sensitivity	Doll et al. (2017), Yuan et al. (2016)
NRF2	Antioxidant response genes	Redox homeostasis	Regulates antioxidant defenses	Associated with poor prognosis	He et al. (2020), Serrão et al. (2018)
FSP1	CoQ10 reduction	Non-canonical ferroptosis pathway	Inhibits ferroptosis	Enhances drug resistance	Doll et al. (2019)
NCOA4	Ferritin	Ferritinophagy	Releases intracellular iron	Promotes ferroptosis	Gao et al. (2016), Hou et al. (2016)
TFRC (TFR1)	Transferrin-bound iron	Iron uptake	Supports proliferation and ferroptosis	Facilitates tumor growth	Chen et al. (2020), Levy et al. (1999)
SLC40A1 (Ferroportin)	Iron efflux	Iron homeostasis	Prevents iron overload	Inhibits ferroptosis	Geng et al. (2018), Shang et al. (2020)
LOX (Lipoxygenases)	PUFA oxidation	Lipid peroxidation	Promotes oxidative stress	Increases ferroptosis sensitivity	Kuhn et al. (2015), Hinman et al. (2018)
BH4 (Tetrahydrobiopterin)	Phospholipid protection	Antioxidant activity	Counters lipid peroxidation	Reduces ferroptosis	Kraft et al. (2019)

### 2.1 Iron and ferroptosis

Having too much iron is a key part of making ROS through enzyme activity and the Fenton reaction, which is linked to the peroxidation of lipids. Iron is essential for physiological activities, although too much iron can cause ferroptosis. Factors in iron metabolism—including iron intake, storage, use, and release—closely control ferroptosis (Chen et al., 2020).

In pulmonary tissues, iron can also come from inhaled particles—such as mining dust, cigarette smoke, and pollutants—or from the breakdown of red blood cells (Ali et al., 2017). Alveolar macrophages (AMs) and alveolar epithelial cells (AECs) take in this iron via transferrin receptor 1 (TFR1) and divalent metal transporter 1 (DMT1), then store it in ferritin (Ftn) (Sun et al., 2024). Under normal conditions, this system keeps iron levels in check; however, chronic exposure to external iron sources or disruptions in iron balance can lead to excess iron within the lungs. Such overload amplifies oxidative stress and lipid peroxidation, ultimately leading ferroptotic cell death in respiratory tissue (Xu et al., 2021). The resulting ferroptosis-induced cell death promotes a pro-inflammatory environment, tissue remodeling, and the release of damage-associated molecular patterns (DAMPs), creating a favorable niche for tumor initiation and progression. These processes enhance the susceptibility of lung tissues to carcinogenesis by fostering chronic inflammation and oxidative damage (Firouzjaei and Mohammadi-Yeganeh, 2024).

Iron consumption mediated by serotransferrin or lactotransferrin increases ferroptosis by means of the transferrin receptor (TFRC) (Wang et al., 2020). Upregulating TFRC expression allows oncogenic MYCN to promote iron accumulation. Ferritinophagy, also known as ferritinophagy, is the mechanism by which the cargo receptor NCOA4 triggers autophagy to break down ferritin, thereby encouraging ferroptosis (Gao et al., 2016). On the other hand, iron release linked with the solute carrier family 40 member 1 (SLC40A1) prevents ferroptosis (Geng et al., 2018). While ferritin export prevents ferroptosis, high intracellular iron content resulting from ferritin breakdown increases it (Hou et al., 2016).

Through upregulation of the ferroportin gene (SLC40A1) and ferritin genes (*F*th1 and Flt1), the transcription factor BACH1 can lower iron accumulation, therefore helping to prevent ferroptosis (Nishizawa et al., 2020). Negative regulation of ferroptosis is achieved by certain mitochondrial proteins associated with iron metabolism, including CISD1, CISD2, ISCU, and NFS1 (Zuo et al., 2022). Accumulation of iron is also controlled by specific signaling mechanisms. A transcriptional regulator, Nuclear protein 1 (NUPR1), inhibits cell death caused by ferroptosis and decreases the accumulation of iron by promoting the synthesis of LCN2, the iron transporter (Liu et al., 2021). Iron chelators and antioxidants are capable of preventing ferroptosis, according to recent studies (Imoto et al., 2018; Park and Chung, 2019).

#### 2.1.1 Key targets of iron metabolism in ferroptosis

##### 2.1.1.1 Transferrin receptor (TFRC/TFR1)

Transferrin is a vital glycoprotein that carries iron, primarily in its ferric state (Fe³⁺), through the bloodstream to cells across the body, preventing the potential harm of iron overload. On the cell membrane, the TFRC binds to iron-laden transferrin and then internalizes this complex via receptor-mediated endocytosis. Within the acidic confines of the endosome, iron is liberated and reduced to its ferrous form (Fe^2^⁺), which then passes into the cytoplasm through specific transport channels. Once iron is released, both transferrin and TFRC cycle back to the cell exterior to repeat this process, maintaining iron balance (Levy et al., 1999).

Ferroptosis is a regulated form of cell demise that hinges on iron-fueled oxidative stress and lipid peroxidation. When transferrin and TFRC heighten intracellular iron levels, the accompanying surge in free iron can accelerate the Fenton reaction, producing ROS species that damage membranes and drive cells toward ferroptosis (Chen et al., 2020). Cancer cells, which frequently upregulate TFRC to sustain their rapid proliferation, may be especially susceptible to treatments that leverage this pathway (Drecourt et al., 2018).

##### 2.1.1.2 Solute carrier family 40 member 1 (SLC40A1)

SLC40A1, also known as ferroportin or FPN1, serves as the sole transmembrane exporter of non-heme iron in mammalian cells. Its activity is largely governed by hepcidin, a hormone released via the liver, binding to SLC40A1 and prompts its internalization and eventual breakdown (Song et al., 2022). Once Fe^2^⁺ exits the cell via SLC40A1, it generally requires oxidation by ferroxidases like ceruloplasmin (CP) so that Fe³⁺ can be loaded onto transferrin (TF) (Nishizawa et al., 2020). Genetic variations in SLC40A1 can lead to an autosomal dominant form of hemochromatosis (ferroportin disease). For instance, the N144D substitution is associated with iron buildup in tissues and liver cirrhosis. Notably, two additional changes at that same residue (N144H and N144T) can also disrupt SLC40A1 function (Arden et al., 2003).

Rodent models lacking SLC40A1 fail to survive past embryonic stages, whereas selective removal of this transporter in the intestinal tract causes severe iron deficiency (Donovan et al., 2005). Mutations in SLC40A1 can cause autosomal dominant hemochromatosis, leading to iron overload conditions (Njajou et al., 2001). SLC40A1 downregulation promotes ferroptosis, while its overexpression or induction by ponasterone can inhibit this process (Geng et al., 2018).

##### 2.1.1.3 Nuclear receptor coactivator 4 (NCOA4)

NCOA4 drives the selective autophagic breakdown of ferritin, a process called ferritinophagy. Similar to depleting ATG5 or ATG7, silencing NCOA4 itself halts ferritin turnover and reduces erastin-triggered ferroptosis in fibroblasts and pancreatic cancer cells. In contrast, raising NCOA4 levels promotes ferroptosis by accelerating ferritin breakdown, establishing a direct link among ferroptosis and autophagy (Hou et al., 2016). Ferritinophagy is further crucial for ferroptotic death under conditions of cystine deprivation, as indicated by evidence that knocking down ATG3, ATG13, BECN1, or MAP1LC3B mitigates cystine starvation- or erastin-triggered ferroptosis through reducing iron buildup (Gao et al., 2016).

NCOA4-orchestrated ferritinophagy has additionally been implicated in cigarette smoke-induced ferroptosis in lung epithelial cells, hinting at ferroptosis involvement in cigarette smoke-induced chronic obstructive pulmonary disease (Yoshida et al., 2019). Heightened ferritinophagy-mediated iron accumulation has also been tied to the aging process and to carbon tetrachloride (CCl4)-elicited liver fibrosis in murine studies (Kong et al., 2019). These findings underscore the importance of ferritinophagy in ferroptosis across different pathologies. Notably, dihydroartemisinin-triggered ferroptotic cell death can proceed through lysosomal ferritin breakdown even in the absence of standard autophagy, pointing to an alternative route for ferritin degradation during ferroptosis (Chen et al., 2006).

### 2.2 Lipid peroxidation and ferroptosis

The stability of the cell’s outside and interior depends on the cell membrane, which is mainly made up of proteins, lipids, and carbohydrates, regulating the flow of substances and enabling cell communication (Zalba and Ten Hagen, 2017). Lipid peroxide (LPO) is formed when oxygen-free radicals react with polyunsaturated fatty acids (PUFA), typically found in very low levels under normal conditions (Wymann and Schneiter, 2008). However, elevated levels of ROS within cells can interact with PUFA side chains in enzymes, membrane receptors, or phospholipids, resulting in lipid peroxidation (Li and Li, 2020). This process produces significant amounts of LPO, disrupting the cell membrane’s fluidity and permeability, altering cellular function and structure, and ultimately inducing cell death (Gaschler and Stockwell, 2017).

Important enzymes in the metabolism of lipids that play a crucial role in ferroptosis are acyl-coa synthetase long-chain family member 4 (ACSL4), arachidonic acid lipoxygenase 15 (ALOX15), and lysophosphatidylcholine acyltransferase 3 (LPCAT3), (Kuwata and Hara, 2019; Wymann and Schneiter, 2008). ACSL4 catalyzes the formation of acyl Co-A derivatives from adrenoyl (AdA) and acyls-arachidonoyl (AA) (Dixon et al., 2015). Subsequently, these derivatives are incorporated into phosphatidylethanolamines (AA-PE and AdA-PE) through esterification mediated by LPCAT3 (Singh and Rao, 2019). Lipoxygenases (LOX) are a group of enzymes that include iron and are responsible for oxidizing PUFA through the use of enzyme kinase G2 (PHKG2)-dependent iron pools. ALOX15 enzymatically oxidizes AA-PE and AdA-PE, leading to the production of LPO, which ultimately causes ferroptosis (Singh and Rao, 2019).

Additionally, cytochrome B5 reductase (CYB5R1) and cytochrome P450 reductase (POR) contribute to the process of ferroptosis by accelerating the peroxidation of phospholipids (Zou et al., 2020b). Peroxidase (POR) may increase the process of lipid peroxidation by speeding up the conversion among Fe2+ and Fe3+ in heme. Both POR and CYB5R1 have the ability to transport electrons from NAD(P)H to oxygen. This process results in the generation of hydrogen peroxide, which then forms reactive hydroxyl radicals by the Fenton reaction. These radicals promote the production of lipid peroxide (LPO) and cause damage to the integrity of the cell membrane (Yan B. et al., 2021). Despite these findings, the precise mechanisms involved require further investigation.

#### 2.2.1 Key targets of lipid metabolism in ferroptosis

##### 2.2.1.1 Glutathione peroxidase 4 (GPX4)

GPX4 is a critical enzyme in lipid metabolism, functioning as a selenocysteine-containing reductase. By utilizing glutathione (GSH), GPX4 prevents the oxidation of lipid chains and diminishes peroxides in complex lipids such as, phospholipids, cholesterol, and fatty acids, thereby stabilizing hydroxyl lipids (Imai et al., 2017). The availability of GSH is a significant factor in regulating GPX4 activity. Additionally, research has indicated that GPX4 expression is adjusted by the Nrf2 gene and sec-tRNA (Serrão et al., 2018; Stockwell et al., 2017).

##### 2.2.1.2 Lipoxygenase (LOX)

LOX enzymes, which are non-heme iron-containing proteins, Promote the oxidation of unsaturated fatty acids within the plasma membrane, thereby triggering ferroptosis (Kuhn et al., 2015). Key members of the family of LOX consist of LOX5, LOX15, and LOX12. LOX15 and LOX12 are capable of directly phospholipids oxidizing on the plasma membrane, whereas LOX5 requires pre-esterification by phospholipase A2 to do so (Rådmark et al., 2015). Studies have shown that vitamin E can prevent ferroptosis, likely due to its ability to inhibit LOX activity (Hinman et al., 2018; Kagan et al., 2017).

##### 2.2.1.3 Acyl-CoA synthetase long-chain family member 4 (ACSL4)

ACSL4, a constituent of the Acyl CoA synthetase family, participates in the transformation of extended-chain fatty acids into acyl-CoA, serving a crucial function in lipid metabolism (Mashima et al., 2009). In mammals, ACSL4 is one of five subtypes and is essential for long-chain polyunsaturated fatty acid-CoA (PUFA-CoA) synthesis. Elevated expression of ACSL4 promotes the accumulation of long-chain PUFA-CoA and the oxidation of phospholipids in the plasma membrane, enhancing cancer cells' vulnerability to ferroptosis (Doll et al., 2017; Yuan et al., 2016).

##### 2.2.1.4 Lysophosphatidyl transferases (LPCATs)

Esterification, a crucial step in phospholipid peroxidation, is primarily facilitated by lysophosphatidyl transferases (LPCATs) (Wang and Tontonoz, 2019). These enzymes are vital for phospholipid remodeling within the endoplasmic reticulum, attaching fatty acyl chains to the sn-2 position of phosphatidylcholine and thereby regulating phospholipid composition. Long-chain PUFAs esterify lysophospholipids through LPCATs following CoA linkage by ACSL4, triggering peroxidation and causing ferroptosis (Conrad et al., 2018). LPCAT3 is critically involved in lipid metabolism, where it regulates processes such as lipid uptake, lipoprotein secretion, and *de novo* lipogenesis (Wang and Tontonoz, 2019).

### 2.3 ROS and ferroptosis

Glutathione (GSH), composed of glutamate (Glu), glycine (Gly), and cysteine (Cys), serves as a powerful antioxidant and free radical scavenger. The cystine/glutamate antiporter system (system xc−), present on the plasma membrane, includes a heavy chain (4F2) and a light chain (xCT) (Lv et al., 2019). This system exports glutamate while importing cystine (Cys2) in a 1:1 ratio, which is then reduced to cysteine (Cys) within the cell, facilitating GSH synthesis (Conrad and Sato, 2012). GPX4 plays a pivotal role in the glutathione antioxidant system by using GSH to alter lipid peroxide (LPO) into non-toxic lipid alcohols, thereby protecting the cell membrane from ROS-induced lipid peroxidation (Imai et al., 2017).

The production of GSH is strongly controlled via system xc−. When cystine is scarce and cysteine uptake is obstructed, GSH synthesis decreases, leading to the buildup of lipid peroxides and the onset of ferroptosis. Disrupting GPX4, preventing system xc−, or halting the synthesis of GPX4 disrupts the cellular redox balance, reducing antioxidant defenses and hindering the elimination of lipid peroxides (Kong et al., 2021).

#### 2.3.1 Key targets of ROS in ferroptosis

##### 2.3.1.1 SLC7A11 (solute carrier family 7 member 11)

SLC7A11, additionally referred to as xCT, is a constituent of the cystine/glutamate antiporter system Xc-. Its primary function is to preserve cellular redox equilibrium by enabling the transport of cystine into cells while simultaneously exchanging it for glutamate. SLC7A11 imports cystine, which is essential for the production of glutathione (GSH), a primary antioxidant that counteracts ROS. SLC7A11 protects cells from lipid oxidation and peroxidation damage by maintaining high levels of GSH, thereby preventing ferroptosis. Increasing or decreasing the activity of SLC7A11 stops the production of GSH, which makes cells more vulnerable to ferroptosis (Koppula et al., 2021).

##### 2.3.1.2 Ferroptosis suppressor protein 1 (FSP1)

Non-canonical pathways also influence ferroptosis regulation. FSP1 operates concurrently with GPX4 by converting coenzyme Q10 (CoQ10) to ubiquinol (CoQH2) at the cell membrane, which then prevents ferroptosis via capturing free radicals (Doll et al., 2019; Imai et al., 2017). FSP1 also decreases vitamin K to hydroquinone, offering additional protection against ferroptosis (Mishima et al., 2022).

##### 2.3.1.3 Nuclear factor erythroid 2–related factor 2 (Nrf2)

NRF2 serves as a key regulator of cellular antioxidant systems and redox balance. Under conditions of oxidative or electrophilic stress, NRF2 separates from KEAP1, moves into the nucleus, and activates the transcription of vital antioxidant enzymes, including superoxide dismutase (SOD), catalase (CAT), glutathione peroxidase (GPx), and heme oxygenase-1 (HO-1), along with additional protective molecules. This gene induction offsets the damaging impact of ROS, thereby helping maintain homeostasis in non-pathological contexts (Turell et al., 2020). Activation of NRF2 increases the cell’s ability to deal with oxidative stress and inhibits ferroptosis. On the other hand, if the NRF2 function is compromised, cells become more susceptible to damage caused by ROS and ferroptosis (He et al., 2020). However, NRF2’s role becomes increasingly complex during cancer development. In the early stages of tumorigenesis, NRF2 activation serves a protective role by mitigating oxidative stress and preventing DNA damage, thereby inhibiting malignant transformation. However, as the tumor progresses to more advanced and metastatic stages, NRF2 activity is often upregulated to support cancer cell survival, enhance resistance to chemotherapy, and facilitate metastatic dissemination (Zimta et al., 2019). This dual role makes NRF2 a complex but promising therapeutic target. Understanding the temporal dynamics of NRF2 can aid in identifying optimal intervention points, either by inhibiting its pro-tumorigenic functions in advanced cancers or by enhancing its protective roles in early stages as a preventive strategy. Although NRF2 safeguards redox balance in normal tissues, its persistent upregulation in malignancies may hasten disease progression (Sekhar and Freeman, 2015). Further research is necessary to pinpoint the exact stages of tumor development where NRF2 activity peaks, which could lead to optimized therapeutic interventions that either enhance its protective functions early on or inhibit its tumorigenic effects in advanced stages.

##### 2.3.1.4 Tetrahydrobiopterin (BH4)

Tetrahydrobiopterin (BH4), produced from guanosine triphosphate (GTP) through a process regulated by the rate-limiting enzyme GTP cyclohydrolase 1 (GCH1), possesses inherent antioxidant capabilities. BH4 mitigates ferroptosis by specifically protecting phospholipids containing two polyunsaturated fatty acyl chains (Kraft et al., 2019).

These findings indicate that ferroptosis is regulated through multiple pathways beyond those dependent on GSH (Vasquez-Vivar et al., 2022). Proteins like FSP1, DHODH, and molecules like BH4 that work as antioxidants without GSH play important roles. This suggests that there are still a lot of regulatory pathways in ferroptosis that have not been found yet.

## 3 Role of non-coding RNAs in ferroptosis and lung cancer

Ferroptosis is now recognized as a critical type of regulated cell death, uniquely defined by its reliance on iron-driven lipid peroxidation and differing significantly from apoptosis and other cell death mechanisms (Ghafouri-Fard et al., 2020). Mounting evidence indicates that ncRNAs—including miRNAs, lncRNAs, and circRNAs—play key regulatory roles in this process by modulating iron homeostasis, lipid metabolism, and redox balance (Hazari et al., 2024). In lung cancer, ncRNAs can influence tumor progression, drug resistance, and clinical outcomes by affecting ferroptosis (Ishola et al., 2020). Consequently, this section intends to present a thorough overview of how various ncRNAs contribute to ferroptosis regulation and shape the pathogenesis and treatment response of lung cancer.

### 3.1 Role of miRNAs in regulating ferroptosis in lung cancer

MicroRNAs are a subclass of non-coding RNAs, typically 21–25 nucleotides in length and are abundant and highly conserved across species. Initially discovered by Lee et al., miRNAs are pivotal in regulating gene transcription and translation (Lee et al., 2004). They play a broad role in physiological processes, pathological conditions, and tumor metabolism. Recent studies have identified numerous miRNAs that regulate ferroptosis through various pathways (Rezaei and Sadri, 2021). Recent research has increasingly displayed the crucial role of miRNAs in modulating ferroptosis by interacting with genes associated with this process. For instance, miR-214 has been recognized as a suppressor of TFRC, thereby mitigating ferroptosis (Lu et al., 2020). Similarly, miR-424-5p and miR-4291 downregulate ACSL4, effectively inhibiting ferroptosis (Lin-Lin et al., 2021; Ou et al., 2022). Alternatively, miR-124 and miR-30b-5p promote ferroptosis by targeting FPN1 (Bao et al., 2020; Zhang et al., 2020). Additionally, miR-541-3p and miR-324-3p enhance ferroptosis by inhibiting GPX4 (Xu et al., 2020; Yu et al., 2022), while miR-375, miR-128-3p, and miR-27a-3p contribute to ferroptosis induction by downregulating SLC7A11 (Lu et al., 2021b; Ni et al., 2021; Zhang et al., 2021).

In lung cancer, these miRNAs are crucial as they can modulate ferroptosis, thereby influencing cancer cell survival and response to therapies (Lu et al., 2021a). Understanding the specific miRNAs and their mechanisms can provide insights into new therapeutic strategies for lung cancer. miR-324-3p is significantly downregulated in cisplatin-resistant A549 cells (A549/DDP), and its overexpression enhances cisplatin sensitivity by targeting and inhibiting GPX4. This inhibition promotes ferroptosis, which is mimicked by the GPX4 inhibitor RSL3, suggesting that targeting the miR-324-3p-GPX4 axis could effectively counteract cisplatin resistance in NSCLC (Deng et al., 2021).

One important study investigated the role of exosomal miR-4443 in enhancing resistance to cisplatin in NSCLC by controlling FSP1-mediated ferroptosis. Exosomes from NSCLC tissues that were resistant to cisplatin had high levels of miR-4443. This miR-4443 gave cells that were sensitive to cisplatin resistance by stopping FSP1-induced ferroptosis. Bioinformatics and luciferase assays confirmed that METTL3 is a direct target of miR-4443, linking miR-4443 to FSP1 regulation via m6A modification. These findings suggested that targeting the miR-4443/METTL3/FSP1 axis could be a potential therapeutic strategy to overcome cisplatin resistance in NSCLC by inducing ferroptosis (Song et al., 2021).

The use of MiR-302a-3p mimics led to increased iron overload, elevated lipid peroxidation, and induced ferroptosis, thereby hindering the growth of NSCLC cells. Conversely, miR-302a-3p inhibitors blocked ferroptosis induced by erastin or RSL3. miR-302a-3p was found to reduce ferroportin (SLC40A1) expression by binding to its 3′-untranslated region. Overexpressing SLC40A1 countered miR-302a-3p-induced ferroptosis. Moreover, miR-302a-3p increased the sensitivity of NSCLC cells to cisplatin and paclitaxel, suggesting its role as a tumor suppressor by inducing ferroptosis via SLC40A1 targeting (Wei et al., 2021).

Researchers discovered that NSCLC tissues upregulate SLC7A11, an important regulator of ferroptosis, which is associated with a poor prognosis. MiR-27a-3p was identified as a key modulator of ferroptosis by targeting SLC7A11. Upregulation of miR-27a-3p inhibited SLC7A11, reducing ferroptosis, while inhibition of miR-27a-3p increased NSCLC cells' sensitivity to ferroptosis. The study concludes that miR-27a-3p has a critical impact on NSCLC progression by modulating ferroptosis through SLC7A11 (Lu et al., 2021a).

MiR-6077 was found as a key contributor to cisplatin (CDDP) and pemetrexed (PEM) resistance in LUAD using CRISPR-Cas9 screening. Overexpression of miR-6077 was found to desensitize LUAD cells to these chemotherapeutic agents by regulating CDKN1A and KEAP1, which are responsible for inhibiting cell-cycle arrest and ferroptosis pathways, respectively (Bi et al., 2022).

Furthermore, the study found that propofol decreases cisplatin resistance in NSCLC by inducing GPX4-mediated ferroptosis via the miR-744-5p/miR-615-3p axis. The study showed that propofol increased miR-744-5p and miR-615-3p expression, which inhibited GPX4 transcription. This upregulation led to increased ferroptosis and reduced cisplatin chemoresistance in NSCLC cells. Additionally, *in vivo* experiments demonstrated that propofol suppressed tumor growth and decreased cisplatin resistance by enhancing the expression of these microRNAs and inhibiting GPX4, reinforcing its potential as a therapeutic option for NSCLC (Han et al., 2023).

Moreover, miR-186-5p was found to be markedly suppressed in LUAD. The overexpression of miR-186-5p significantly downregulated both gene and protein expression of PRKAA2, thereby promoting ferroptosis and effectively inhibiting LUAD cell proliferation, invasion, and migration. In addition, miR-186-5p elevated ROS levels in LUAD cells. Furthermore, the combined effect of miR-186-5p overexpression and PRKAA2 silencing increased MDA content while reducing GSH levels, enhancing ferroptosis sensitivity by specifically targeting PRKAA2. Therefore, miR-186-5p was shown to inhibit LUAD cellular proliferation, migration, and invasion through the targeted regulation of PRKAA2, promoting ferroptosis (Liu L. et al., 2023). Furthermore, miRNA34a combined with Jurkat T cells was studied for its effects on NSCLC. It was found that the ferroptosis pathway contributed to the killing of A549 cancer cells, particularly due to the presence of miRNA34a and iron oxide nanorods (IONRs) as the delivery agents (Pandey et al., 2024).

The EGR1/miR-139/NRF2 axis was found to influence radiosensitivity in NSCLC through ferroptosis. MiR-139 acted as a radiosensitizer by inhibiting NRF2 signaling, enhancing IR-induced peroxidation of lipid and ferroptosis in NSCLC cells. MiR-139 targeted c-JUN and KPNA2 directly, with KPNA2 facilitating NRF2’s nuclear translocation. Ionizing radiation induced miR-139 expression via the transcription factor EGR1, which bound to miR-139s promoter. These findings highlight the potential of the EGR1/miR-139/NRF2 axis as a diagnostic therapeutic target and biomarker for improving radiosensitivity in NSCLC (Zhang et al., 2024b).

Interestingly, the compound Sanggenol L was discovered to trigger ferroptosis in NSCLC cells via activating the miR-26a-1-3p/MDM2/p53 signaling pathway. The occurrence of this process was demonstrated by a rise in the buildup of ROS, a decrease in glutathione levels, shrinkage of mitochondria, and peroxidation of lipids.

**FIGURE 2:**
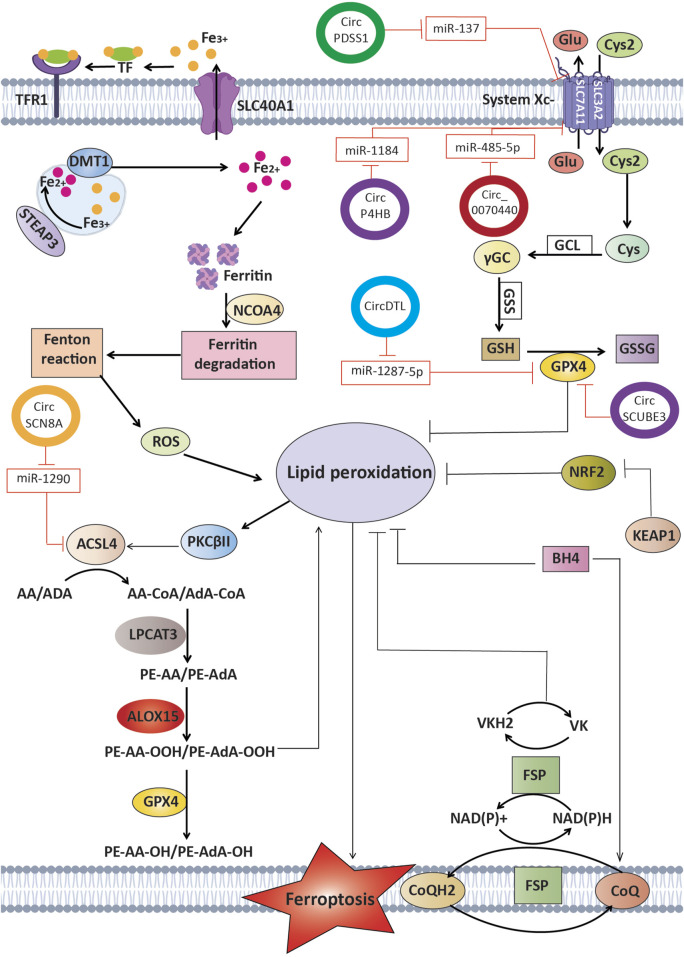
MicroRNAs that specifically target crucial elements of the ferroptosis pathway. This picture illustrates the role of miRNAs in regulating ferroptosis in lung cancer by targeting crucial molecules including GPX4, SLC7A11, ACSL4, and others, hence affecting the peroxidation of lipids, the metabolism of iron, and ROS balance. These miRNAs play a crucial role in either increasing or suppressing ferroptosis, which in turn affects the survival of cancer cells and their response to treatment.

Sanggenol L treatment was found to upregulate miR-26a-1-3p, which directly targets MDM2. This interaction increased p53 protein levels while reducing SLC7A11 expression, thereby triggering ferroptosis. The therapeutic potential and safety profile of sanggenol L were confirmed using subcutaneous xenograft and patient-derived tumor xenograft models. These results underscore the importance of the miR-26a-1-3p/MDM2/p53/SLC7A11 pathway in facilitating sanggenol L-induced ferroptosis, positioning it as a promising therapeutic option for NSCLC (Table 2) (Figure 2) (Fu et al., 2024).

**TABLE 2:** Role of miRNAs in regulating ferroptosis in lung cancer.

miRNA	Expression/Change	Role in ferroptosis	Target(s)	Mechanism in ferroptosis	Therapeutic implications	Ref
miR-324-3p	Downregulated in cisplatin-resistant A549	Promotes ferroptosis	GPX4	Overexpression blocks GPX4, boosting ferroptosis and restoring cisplatin sensitivity	Potential strategy to overcome cisplatin resistance	Deng et al. (2021)
miR-4443	Elevated in exosomes of cisplatin-resistant NSCLC	Inhibits ferroptosis	FSP1 (via METTL3)	Downregulates FSP1-induced ferroptosis by targeting METTL3, conferring cisplatin resistance	Targeting miR-4443/METTL3/FSP1 axis may enhance chemosensitivity	Song et al. (2021)
miR-302a-3p	Overexpression increases iron overload	Promotes ferroptosis	SLC40A1	Reduces SLC40A1 expression, causing iron accumulation and lipid peroxidation	Enhances sensitivity to cisplatin and paclitaxel	Wei et al. (2021)
miR-27a-3p	Upregulated in NSCLC tissues	Inhibits ferroptosis	SLC7A11	Represses ferroptosis by targeting SLC7A11; reducing miR-27a-3p restores ferroptotic response	Could be a therapeutic target to induce ferroptosis	Lu et al. (2021)
miR-6077	Overexpression desensitizes LUAD cells	Inhibits ferroptosis	CDKN1A, KEAP1	Reduces cell-cycle arrest and ferroptosis, contributing to CDDP and PEM resistance	Blocking miR-6077 might reverse chemoresistance	Bi et al. (2022)
miR-744-5p/miR-615-3p	Upregulated by propofol	Promotes ferroptosis	GPX4	Inhibit GPX4 transcription, increasing ferroptosis and reducing cisplatin resistance	Use of propofol-based regimens to potentiate ferroptosis	Han et al. (2023)
miR-186-5p	Downregulated in LUAD	Promotes ferroptosis	PRKAA2	Suppresses PRKAA2, driving ROS accumulation and ferroptotic cell death	May serve as a therapeutic tool to limit LUAD progression	Liu et al. (2023b)
miR-34a	Combined with Jurkat T cells, tested in A549	Promotes ferroptosis	Not specified	Induces ferroptotic killing of A549 cells, particularly with iron oxide nanorods	Potential ferroptosis-based immunotherapy approach	Pandey et al. (2024)
miR-139	Upregulated by IR via EGR1	Promotes ferroptosis (radiosensitizer)	NRF2 (via cJUN, KPNA2)	Inhibits NRF2 activation, enhancing IR-triggered lipid peroxidation and ferroptosis	Could improve radiosensitivity in NSCLC	Zhang et al. (2024b)
miR-26a-1-3p	Elevated upon Sanggenol L treatment	Promotes ferroptosis	MDM2 → p53 → SLC7A11	Targets MDM2, raising p53 levels and downregulating SLC7A11, stimulating ferroptosis	Potential therapeutic axis for NSCLC management	Fu et al. (2024)

In summary, these findings demonstrate that a variety of miRNAs can modulate ferroptosis through direct targets such as GPX4, FSP1, and SLC7A11, or via upstream signaling pathways in lung cancer. By fine-tuning ferroptotic sensitivity, miRNAs not only influence tumor cell survival but also impact the efficacy of chemotherapeutic agents. Therefore, targeting miRNA expression through therapeutic approaches could be a promising strategy to combat drug resistance and enhance treatment outcomes in lung cancer patients.

### 3.2 Role of lncRNAs in regulating ferroptosis in lung cancer

LncRNAs, typically over 200 nucleotides long, regulate gene expression at multiple levels and interact with DNA, mRNA, miRNA, and proteins to influence various cellular pathways (Statello et al., 2021). For instance, lncRNA PVT1 (lncPVT1) has been shown to suppress miR-214, thereby upregulating TFRC and p53, which contributes to ferroptosis induction in brain ischemia/reperfusion (I/R) injury (Lu et al., 2020). Similarly, lncOIP5-AS1 inhibits miR-128-3p, resulting in increased SLC7A11 expression and the suppression of ferroptosis in prostate cancer (Zhang et al., 2021). Moreover, lncRNAs have been recognized as key regulators of ferroptosis by interacting with proteins, affecting mRNA stability and protein ubiquitination. For instance, lncPMAN interacts with ELAVL1, stabilizing SLC7A11 mRNA, thereby inhibiting ferroptosis (Lin et al., 2022). In contrast, lncHEPFAL facilitates the ubiquitination and degradation of the SLC7A11 protein, thereby promoting ferroptosis (Zhang B. et al., 2022). In addition, lncP53RRA binds to Ras GTPase-activating protein-binding protein 1 (G3BP1), facilitating the retention of p53 in the nucleus and promoting ferroptosis (Mao et al., 2018; Zhang B. et al., 2022).

In lung cancer, they modulate ferroptosis, thereby affecting tumor cell survival, proliferation, and treatment responses, which underscores their potential as therapeutic targets (Guo et al., 2021). Long noncoding RNA LINC00336, upregulated in LC, inhibits ferroptosis by acting as a competing endogenous RNA (ceRNA) for miR-6852, thereby regulating cystathionine-β-synthase (CBS) expression. It binds to RNA-binding protein ELAVL1, which stabilizes LINC00336 via the p53 signaling pathway, promoting the LINC00336/miR-6852/CBS axis and contributing to ferroptosis inhibition and tumorigenesis in lung cancer (Pandey et al., 2024; Wang et al., 2019a; Wang et al., 2019b). RNA sequencing of XAV939-treated and untreated NCI-H1299 cells identified numerous differentially expressed lncRNAs and genes involved in ferroptosis, particularly targeting SLC7A11. CeRNA network analysis revealed critical interactions, such as the downregulation of lncRNA MIR503HG, which sponges miR-1273c to regulate SOX4 expression. This indicates that XAV939 may inhibit NSCLC progression by modulating ferroptosis through lncRNA-miRNA-mRNA interactions (Yu et al., 2019).

Curcumenol, a component derived from Wenyujin, downregulates long non-coding RNA H19 (lncRNA H19), triggering ferroptosis by enhancing miR-19b-3p activity and suppressing its target gene, ferritin heavy chain 1 (FTH1). Conversely, upregulation of lncRNA H19 counteracts curcumenol’s anticancer effects, while its knockdown amplifies ferroptosis. These findings suggest that curcumenol combats tumors through the lncRNA H19/miR-19b-3p/FTH1 axis, presenting a potential strategy for the treatment of LC (Zhang R. et al., 2022). A bioinformatic study explored the ferroptosis-related lncRNA GSEC/miRNA-101-3p/CISD1 axis in LUAD, identifying upregulated genes like CISD1, PGD, and ATP5MC3 associated with advanced tumor stages and poor prognosis. The GSEC/miRNA-101-3p axis is crucial for LUAD progression, as knocking down CISD1 significantly inhibits cellular proliferation and migration. These findings suggest that the GSEC/miRNA-101-3p/CISD1 axis could serve as a biomarker for LUAD prognosis and a potential therapeutic target (Jiang X. et al., 2022). Additionally, lncRNAs GMDS-AS1 and LINC01128 enhance LUAD cell sensitivity to CDDP/PEM by sponging miR-6077, indicating that targeting miR-6077 may overcome chemoresistance in LUAD (Bi et al., 2022).

Bioinformatic analysis identified the miR-17-5p/HOXA7 axis as crucial in LUAD with brain metastasis related to ferroptosis, highlighting the HCP5/hsa-miR-17-5p/HOXA7 ceRNA network. A significant correlation between HOXA7 and the ferroptosis index suggests that HCP5 competitively interacts with miR-17-5p to enhance HOXA7 expression, thereby inhibiting brain metastasis. This ceRNA axis may significantly influence LUAD brain metastasis development (Chen et al., 2022).

The miR-299-3p/SLC38A1 axis has revealed OGFRP1 as a key regulator of cellular proliferation and ferroptosis in lung cancer. While OGFRP1 and SLC38A1 are increased in LC tissues and cells, miR-299-3p is significantly downregulated. Silencing OGFRP1 suppresses cell proliferation and induces ferroptosis through increasing the accumulation of iron and the peroxidation of lipid. Conversely, inhibiting miR-299-3p or overexpressing SLC38A1 reverses the effects of OGFRP1 knockdown on cell growth and ferroptosis. Furthermore, OGFRP1 deficiency reduces tumor growth in animal models, highlighting its potential as a therapeutic target in lung cancer treatment (Liu et al., 2022).

Cinobufotalin inhibits the proliferation of lung cancer cells by activating ferroptosis through the LINC00597/hsa-miR-367-3p/TFRC signaling pathway. It increases LINC00597 expression while suppressing hsa-miR-367-3p levels in lung cancer cells, mediated by resibufogenin. TFRC, a key promoter of ferroptosis, is negatively regulated by hsa-miR-367-3p, and LINC00597 enhances TFRC expression by sequestering hsa-miR-367-3p. These findings suggest that Cinobufotalin may offer novel targets for lung cancer treatment through this pathway (Huang et al., 2023). LINC00551 exhibits low expression in LUAD and other malignancies. Its overexpression reduces cell survival and enhances autophagy and RSL-3-induced ferroptosis in LUAD cells by acting as a ceRNA for miR-4328, leading to increased DNA damage-inducible transcript 4 (DDIT4) expression. DDIT4 suppresses mTOR activity, stimulates autophagy, and triggers ferroptosis, highlighting LINC00551 as a promising therapeutic target for LUAD (Peng et al., 2022). LncRNAITGB2-AS1 is elevated in NSCLC patients and cisplatin-resistant NSCLC cells. Knocking down ITGB2-AS1 inhibits cell growth and enhances apoptosis and ferroptosis by downregulating NAMPT expression through interaction with FOSL2, leading to the suppression of p53. *In vivo*, ITGB2-AS1 inhibition reduces cisplatin resistance, indicating that ITGB2-AS1 promotes cisplatin resistance in NSCLC by blocking p53-mediated ferroptosis via the FOSL2/NAMPT pathway (Chen et al., 2023).

SDCBP2-AS1 expression is increased in lung cancer cells, and its downregulation enhances ferroptosis. Acting as a miR-656-3p decoy, SDCBP2-AS1 sequesters miR-656-3p, preventing it from targeting CRIM1. Rescue experiments demonstrated that SDCBP2-AS1 regulates ferroptosis by modulating the miR-656-3p/CRIM1 axis, with elevated CRIM1 levels counteracting miR-656-3p′s (Dai et al., 2023). Long noncoding RNA NEAT1 plays a significant role in ferroptosis sensitivity and drug resistance in non-small-cell lung cancer. NEAT1 regulates ferroptosis by targeting ACSL4 and modulating pathways involving GPX4 and SLC7A11. Silencing NEAT1 increases ferroptosis sensitivity in NSCLC cells, suggesting potential therapeutic strategies. Additionally, the NEAT1_1/miR-338-3p/AKR1C1 pathway is implicated in gefitinib resistance in EGFR-mutated lung adenocarcinoma, where increased AKR1C1 expression enhances defense against ferroptosis. These findings highlight the critical pathways through which NEAT1 regulates ferroptosis and contributes to therapeutic resistance in lung cancer (Wu and Liu, 2021; Zhen et al., 2024).

Lung adenocarcinoma and lung squamous cell carcinoma tissues exhibit dramatically increased LUCAT1 expression. In A549 cells, LUCAT1 expression is upregulated by RSL3 treatment, an effect counteracted by Fer-1. Elevated LUCAT1 enhances cellular proliferation and suppresses ferroptosis, while its downregulation reduces proliferation and promotes ferroptosis by decreasing GCH1 and FSP1 levels and increasing miR-34a-5p, which downregulates GCH1. Thus, inhibiting LUCAT1 promotes ferroptosis through the miR-34a-5p-mediated GCH1 downregulation, suggesting a potential treatment strategy for lung cancer (Tai et al., 2024). Similarly, overexpression of LncRNA Uc.339 has been documented in lung adenocarcinoma. It has been suggested that LncRNA Uc.339 interacts with pre-miR-339, thereby inhibiting its maturation process. This interaction subsequently leads to an increase in the expression of SLC7A11, a downstream target of miR-339 (Table 3) (Zhang N. et al., 2022).

**TABLE 3:** An overview of the expression changes, roles, targets, mechanisms in ferroptosis, and therapeutic implications for various lncRNAs in lung cancer.

lncRNA	Expression change	Role	Target	Mechanism in ferroptosis	Therapeutic implications	Ref.
LINC00336	Upregulated	Inhibitor of ferroptosis	miR-6852, CBS	Acts as a ceRNA for miR-6852, regulating CBS expression via ELAVL1, impacting p53 signaling	Targeting LINC00336/MIR6852/CBS axis to induce ferroptosis in lung cancer	Pandey et al. (2024), Wang et al. (2019a), Wang et al. (2019b)
MIR503HG	Downregulated	Potential inhibitor	miR-1273c, SOX4	Sponges miR-1273c, regulating SOX4 expression, possibly affecting ferroptosis	XAV939 modulation in NSCLC may alter ferroptosis through lncRNA-miRNA-mRNA interactions	Yu et al. (2019)
H19	Downregulated	Inhibitor of ferroptosis	miR-19b-3p, FTH1	miR-19b-3p activity enhances, suppressing FTH1, triggering ferroptosis	Curcumenol targets H19/miR-19b-3p/FTH1 axis, potential lung cancer therapy	Zhang et al. (2022b)
GSEC	Upregulated in LUAD	Inhibitor of ferroptosis	miRNA-101-3p, CISD1	Regulates ferroptosis-related genes (CISD1), associated with poor prognosis	GSEC/miRNA-101-3p/CISD1 axis as prognostic marker and therapeutic target in LUAD	Jiang et al. (2021)
GMDS-AS1, LINC01128	-	Inhibitor of chemoresistance	miR-6077	Sponges miR-6077, overcoming chemoresistance in LUAD	Targeting miR-6077 to overcome chemoresistance	Bi et al. (2022)
HCP5	Upregulated	Inhibitor of brain metastasis	miR-17-5p, HOXA7	Competes with miR-17-5p, upregulating HOXA7, inhibiting brain metastasis	HCP5/hsa-miR-17-5p/HOXA7 axis in LUAD brain metastasis	Chen et al. (2022)
OGFRP1	Upregulated	Promoter of proliferation	miR-299-3p, SLC38A1	Regulates cell proliferation and ferroptosis through miR-299-3p and SLC38A1	Targeting OGFRP1/miR-299-3p/SLC38A1 axis for lung cancer treatment	Liu et al. (2022)
LINC00597	Upregulated	Promoter of ferroptosis	hsa-miR-367-3p, TFRC	Upregulates TFRC by sponging hsa-miR-367-3p, inducing ferroptosis	Cinobufotalin may offer novel therapeutic targets	Huang et al. (2023)
LINC00551	downregulated	Promoter of ferroptosis	miR-4328, DDIT4	Inhibits mTOR activity, promotes autophagy and ferroptosis in an autophagy-dependent manner	Potential therapeutic target in LUAD	Peng et al. (2022)
ITGB2-AS1	Upregulated	Promoter of chemoresistance	NAMPT, FOSL2, p53	Inhibits p53-mediated ferroptosis through NAMPT expression, binding to FOSL2	ITGB2-AS1 as a target to overcome cisplatin resistance in NSCLC	Chen et al. (2023)
SDCBP2-AS1	Upregulated	Inhibitor of ferroptosis	miR-656-3p, CRIM1	Sponges miR-656-3p, regulates CRIM1, promoting ferroptosis	Targeting SDCBP2-AS1/miR-656-3p/CRIM1 axis for lung cancer treatment	Dai et al. (2023)
NEAT1_1	Upregulated	Promoter of chemoresistance	miR-338-3p, AKR1C1	Promotes ferroptosis defense, leading to gefitinib resistance	Targeting NEAT1_1/miR-338-3p/AKR1C1 axis to overcome gefitinib resistance	Wu and Liu (2021), Zhen et al. (2024)
LUCAT1	Upregulated	Inhibitor of ferroptosis	GCH1, FSP1, miR-34a-5p	Downregulates GCH1 and FSP1, promoting ferroptosis through miR-34a-5p-mediated mechanisms	Inhibiting LUCAT1 to induce ferroptosis and inhibit lung cancer progression	Tai et al. (2024)
Uc.339	Upregulated	Inhibitor of ferroptosis	miR-339, SLC7A11	inds to pre-miR-339, inhibiting its maturation, and upregulates SLC7A11, suppressing ferroptosis	Targeting Uc.339 to promote ferroptosis and inhibit lung adenocarcinoma progression	Zhang et al. (2022a)

In conclusion, lncRNAs such as LINC00336, H19, ITGB2-AS1, and others orchestrate ferroptosis through mechanisms including ceRNA networks, p53 signaling regulation, and modulation of key ferroptotic effectors like SLC7A11 and GPX4. By altering the ferroptotic threshold in lung cancer cells, these lncRNAs influence tumor progression and therapy outcomes. Therefore, targeting lncRNAs that regulate ferroptosis may offer novel strategies to enhance chemotherapy responses and overcome drug resistance in NSCLC. Further studies on targeting lncRNAs in ferroptosis could pave the way for novel therapeutic interventions to overcome chemoresistance and improve outcomes in lung cancer patient.

### 3.3 Role of CircRNAs in regulating ferroptosis in lung cancer

CircRNAs are a recently identified group of natural non-coding RNAs that are produced by the reverse splicing of exons or lariat introns from pre-messenger RNA (mRNA) (Robic et al., 2020). This process results in the formation of a closed loop structure that lacks a 3′poly-(A) tail or 5′cap. Circular RNAs possess a distinctive shape that makes them impervious to degradation by exonucleases (Chen, 2020). As a result, they can maintain stable expression in various tissues and cells. Circular RNAs have become a central focus in biological study due to miRNA binding sites, their stability, and regulatory potential. CircRNAs have been implicated in various conditions, including tumor microenvironment regulation, diabetic vascular complications, and neurodegenerative diseases (Chen, 2020; Chen M. et al., 2021; Tong, 2021).

CircRNAs often function by sequestering miRNAs, acting as RNA sponges to regulate the expression of miRNA target proteins. Through this mechanism, several circRNAs have been implicated in ferroptosis regulation by targeting genes involved in this process. For example, circIL4R suppresses miR-541-3p activity, which enhances GPX4 expression and inhibits ferroptosis in hepatocellular carcinoma (Xu et al., 2020). Similarly, circLMO1 downregulates miR-4291, resulting in elevated ACSL4 levels that induce ferroptosis in cervical cancer (Ou et al., 2022).

Beyond miRNA interactions, circRNAs can also directly bind to proteins to influence ferroptosis. For instance, circ-cIARS binds to ALKBH5, an RNA-binding protein that negatively regulates ferritinophagy and ferroptosis, thereby blocking its activity (Ou et al., 2022). CircEXOC5 stabilizes ACSL4 mRNA by interacting with polypyrimidine tract-binding protein 1 (PTBP1),facilitating ferroptosis in the context of sepsis-induced acute lung injury (Wang W. et al., 2022). Additionally, circRAPGEF5 modulates alternative splicing of TFRC by interacting with RBFOX2, an RNA-binding protein, thus conferring resistance to ferroptosis in endometrial cancer cells (Zhang J. et al., 2022).

Other circRNAs have been recognized as key modulators of ferroptosis through distinct mechanisms. CircST6GALNAC6 interacts with HSPB1, inhibiting its phosphorylation at the Ser-15 site, a modification associated with the cellular response to ferroptosis stress (Wang L. et al., 2022). CircLRFN5 promotes degradation of the PRRX2 protein, leading to the suppression of GCH1 and induction of ferroptosis (Jiang Y. et al., 2022). Moreover, circ101093 enhances the uptake and utilization of arachidonic acid by interacting with FABP3, reducing lipid peroxidation and preventing ferroptosis (Zhang et al., 2022e).

New research shows that circRNAs can control ferroptosis in LC cells. This affects the survival of tumor cells and how well they respond to treatment (Shanshan et al., 2021b). The expression of CircDTL was shown to be increased in both NSCLC tissues and cells, where it functions as an oncogene. Suppression of circDTL resulted in the induction of both programmed cell death and iron-dependent cell death in NSCLC cells. The circDTL/miR-1287-5p/GPX4 axis was responsible for mediating this impact, with GPX4 exerting inhibitory effects on both ferroptosis and apoptosis. Researchers also found that blocking circDTL made NSCLC cells more sensitive to chemotherapeutic drugs and slowed down tumor growth in living things. This shows that circDTL could be used as a therapeutic target for NSCLC treatment (Shanshan et al., 2021a). According to important research, circP4HB plays a crucial regulatory role in LUAD by preventing ferroptosis. CircP4HB controls the miR-1184/SLC7A11 axis and makes more glutathione (GSH) through SLC7A11-mediated glutathione synthesis. This keeps cancer cells from going through ferroptosis. High levels of circP4HB in LUAD decrease ferroptosis and accelerate tumor development. The study’s findings point to circP4HB as a promising new biomarker for the early detection and management of LUAD (Wei et al., 2021). Additionally, researchers discovered that exosomes isolated from LUAD patients prevent ferroptosis by reducing lipid peroxidation. Cir101093 is the intermediary for this effect; it raises FABP3 levels, which in turn lower plasma AA levels by promoting the process of transforming acyls-arachidonoy (AA) to N-arachidonoyl taurine (NAT). These results provide more evidence that therapy based on ferroptosis for LUAD could benefit from focusing on exosomal pathways (Zhang et al., 2022f).

Moreover, circSCN8A was discovered to be under-expressed in both NSCLC tissues and cells. The decreased level of circSCN8A was found to be associated with worse prognosis and aggressive clinicopathological characteristics in individuals with NSCLC. CircSCN8A inhibited cellular proliferation, migration, invasion, and epithelial-mesenchymal transition (EMT), while simultaneously enhancing ferroptosis. In terms of how it worked, circSCN8A competed with miR-1290 as an endogenous RNA (ceRNA), which increased the expression of ACSL4. Suppression of ACSL4 or enhancement of miR-1290 counteracted the promotion of ferroptosis and inhibition of cell proliferation caused by circSCN8A, suggesting that circSCN8A could be a promising target for therapeutic intervention in NSCLC (Liu B. et al., 2023).

Circ_0070440 promoted the advancement of cancer and inhibited a kind of cell death called ferroptosis by acting as a sponge for miR-485-5p, which resulted in an increase in the expression of SLC7A11. Suppression of circ_0070440 hindered the growth of LUAD cells and facilitated programmed cell death and ferroptosis. In addition, there were higher levels of circ_0070440 and SLC7A11, and lower levels of miR-485-5p, in LUAD tumor tissues when compared with normal tissues. The evidence indicates that the circ_0070440/miR-485-5p/SLC7A11 axis has a significant impact on the advancement of LUAD and the occurrence of ferroptosis. This finding suggests that targeting this axis could be a promising approach for diagnosing and treating LUAD (Zhao et al., 2023). Further, the suppression of CircPDSS1 was observed to trigger ferroptosis in NSCLC by regulating the miR-137/SLC7A11/GPX4/GCLC pathway. The expression of CircPDSS1 was increased in NSCLC cells in comparison to normal lung cells. Inhibiting the expression of circPDSS1 resulted in a decrease in cell viability and specifically triggered ferroptosis, as opposed to other forms of cell death. In terms of mechanism, circPDSS1 functioned as a “sponge” for miR-137, which specifically targeted SLC7A11. Moreover, the inhibition of circPDSS1 resulted in the decreased expression of GPX4 and GCLC, indicating that the circPDSS1/miR-137/SLC7A11/GPX4/GCLC pathway could be a promising treatment approach for NSCLC. CircSCUBE3 has been identified as a promoter of ferroptosis in LUAD. It regulates GPX4-mediated GSH synthesis by interacting with CREB, suppressing GPX4 transcription, and enhancing ferroptosis sensitivity. Loss-of-function assays demonstrated that circSCUBE3 deficiency reverses erastin-induced ferroptosis and accelerates tumor progression *in vivo*. These findings underscore the potential of targeting circSCUBE3/CREB/GPX4 as a novel therapeutic strategy for LUAD (Fu and Zhao, 2024).

**FIGURE 3:**
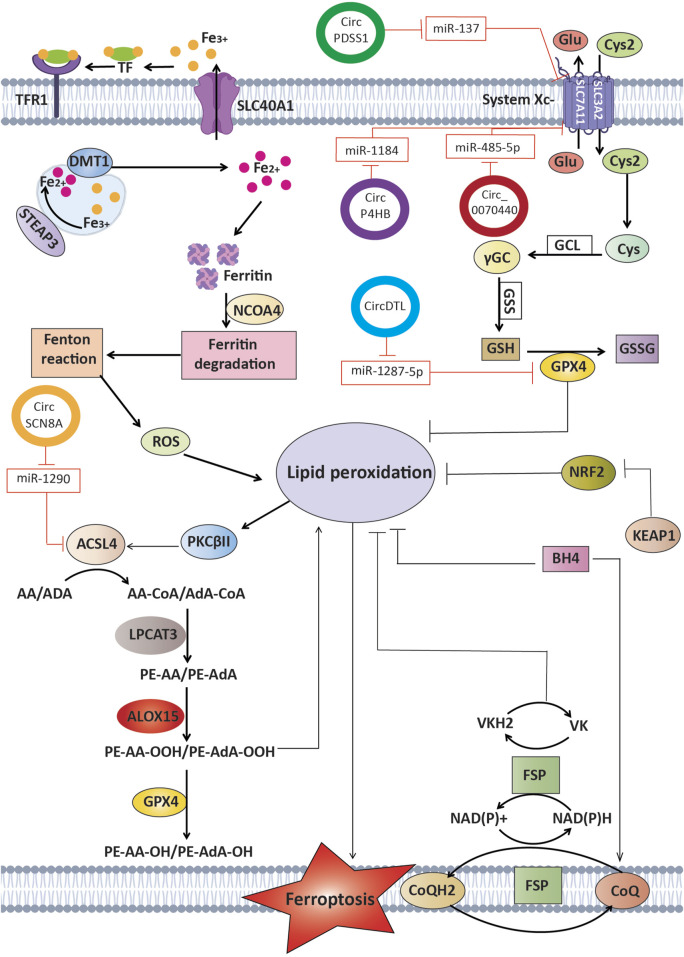
Circular RNAs function as competitive endogenous RNAs by binding and sequestering certain microRNAs that usually target crucial regulators of ferroptosis. This interaction influences processes such as lipid peroxidation and iron metabolism, ultimately affecting the fate of cancer cells and potentially influencing therapy results in lung cancer.

Overall, circRNAs are emerging as vital regulators of ferroptosis in lung cancer by functioning as miRNA sponges (e.g., circDTL/miR-1287-5p/GPX4 axis) and influencing redox homeostasis (Table 4) (Figure 3) (Wu et al., 2024). High or low circRNA expression can either inhibit or promote ferroptotic cell death, thereby affecting tumor growth and therapeutic resistance. Harnessing circRNA-based interventions could therefore provide new avenues for enhancing ferroptosis sensitivity and improving the clinical management of NSCLC.

**TABLE 4:** Regulatory roles and expression changes of circular RNAs (circRNAs) in ferroptosis and lung cancer.

CircRNA	Expression change in cancer	Role	Target/Pathway	Mechanism in ferroptosis	Therapeutic implications	Ref.
CircDTL	Upregulated	Oncogene	miR-1287-5p/GPX4	Promotes ferroptosis and apoptosis	Potential target in NSCLC treatment	Shanshan et al. (2021b)
circP4HB	Upregulated	Protective	miR-1184/SLC7A11	Prevents ferroptosis by increasing GSH synthesis	Early detection and management of LUAD	Pan et al. (2022)
Cir101093	Stable (no change reported)	-	FABP3	Reduces lipid peroxidation	Therapy for LUAD focusing on exosomal pathways	Zhang et al. (2022b)
circSCN8A	Downregulated	Suppresses tumor growth	miR-1290/ACSL4	Enhances ferroptosis sensitivity	Therapeutic intervention in NSCLC	Liu et al. (2023a)
circ_0070440	Upregulated	Promotes cancer	miR-485-5p/SLC7A11	Inhibits ferroptosis	Diagnosis and treatment of LUAD	Zhao et al. (2023)
CircPDSS1	Upregulated	-	miR-137/SLC7A11/GPX4/GCLC	Induces ferroptosis	Promising treatment approach for NSCLC	Wu et al. (2024)
CircSCUBE3	Stable (no change reported)	Promotes ferroptosis	CREB/GPX4	Enhances GSH synthesis and ferroptosis sensitivity	Novel strategy for LUAD	Fu and Zhao (2024)

## 4 Conclusion

ROS The relationship between ferroptosis and ncRNAs represents a highly promising field in lung cancer research, offering fresh perspectives on cancer biology and innovative therapeutic strategies. This review emphasized the critical roles of microRNAs, long noncoding RNAs, and circular RNAs in regulating ferroptosis. NcRNAs are instrumental in managing essential biochemical pathways related to ferroptosis, including iron metabolism, ROS balance, and lipid peroxidation processes. Acting as competitive endogenous RNAs, ncRNAs modulate the expression of pivotal ferroptosis-associated genes such as GPX4, SLC7A11, and ACSL4. For example, miR-324-3p, miR-4443, miR-302a-3p, miR-27a-3p, and miR-186-5p are connected to crucial antioxidant and iron-transport mechanisms, while specific lncRNAs (e.g., LINC00336, ITGB2-AS1, LINC00597) and circRNAs (circDTL, circPDSS1) function through unique miRNA–mRNA interactions.

From a clinical perspective, targeting ncRNAs that inhibit ferroptosis may re-sensitize drug-resistant lung tumors to iron-dependent cell death, thereby reducing therapeutic resistance. On the other hand, regulating ncRNAs that facilitate ferroptosis can protect normal cells from unintended oxidative damage. Leveraging these ncRNA-driven networks, especially in combination with existing treatments, could lead to more personalized and effective approaches in managing lung cancer. Ultimately, interventions centered on ncRNAs hold the potential to enhance ferroptosis in cancerous cells while preserving healthy tissues, marking a significant advancement in the field of precision oncology.

## Glossary

ACSL4: AcylCoA Synthetase LongChain Family Member 4
AKT: Protein Kinase B
ALK: Anaplastic Lymphoma Kinase
ALOX15: Arachidonic Acid Lipoxygenase 15
BCL2: BCell Lymphoma 2
BH4: Tetrahydrobiopterin
CART: Chimeric Antigen Receptor TCell Therapy
CDDP: Cisplatin
CDKs: CyclinDependent Kinases
CircRNAs: Circular RNAs
CP: Ceruloplasmin
CTLA4: Cytotoxic TLymphocyte Associated Protein 4
CXCL: CXC Motif Chemokine Ligand
CYB5R1: Cytochrome B5 Reductase 1
DDP: Cisplatin
DMT1: Divalent Metal Transporter 1
EGFR: Epidermal Growth Factor Receptor
EMT: EpithelialMesenchymal Transition
FSP1: Ferroptosis Suppressor Protein 1
Ftn: Ferritin
GCH1: GTP Cyclohydrolase 1
GCLC: GlutamateCysteine Ligase Catalytic Subunit
GPX4: Glutathione Peroxidase 4
GSH: Glutathione
HIF1α: HypoxiaInducible Factor 1 Alpha
ILs: Interleukins
IONRs: Iron Oxide Nanorods
IR: Ionizing Radiation
JAK/STAT: Janus Kinase/Signal Transducer and Activator of Transcription Pathway
KRAS: Kirsten Rat Sarcoma Viral Oncogene Homolog
LC: Lung Cancer
LCC: Large Cell Carcinoma
LncRNAs: Long Noncoding RNAs
LPCAT3: Lysophosphatidylcholine Acyltransferase 3
LUAD: Lung Adenocarcinoma
LUSC: Lung Squamous Cell Carcinoma
MAPK: MitogenActivated Protein Kinase
MET: MesenchymalEpithelial Transition Factor
MiRNAs: MicroRNAs
MMPs: Matrix Metalloproteinases
MTOR: Mechanistic Target of Rapamycin
NCOA4: Nuclear Receptor Coactivator 4
NcRNAs: Noncoding RNAs
NFκB: Nuclear Factor Kappa B
NRF2: Nuclear Factor Erythroid 2–Related Factor 2
NSCLC: NonSmall Cell Lung Cancer
PCD: Programmed Cell Death
PD1: Programmed Death1
PDL1: Programmed DeathLigand 1
PEM: Pemetrexed
PHKG2: Phosphorylase Kinase G2
PI3K: Phosphoinositide 3Kinase
POR: Cytochrome P450 Reductase
RNS: Reactive Nitrogen Species
ROS: Reactive Oxygen Species
SCLC: Small Cell Lung Cancer
SLC40A1: Solute Carrier Family 40 Member 1
SLC7A11: Solute Carrier Family 7 Member 11
STAT3: Signal Transducer and Activator of Transcription 3
TFR1: Transferrin Receptor 1
TFRC: Transferrin Receptor
TILs: TumorInfiltrating Lymphocytes
TME: Tumor Microenvironment
TNF-α: Tumor Necrosis Factor Alpha
TP53: Tumor Protein P53
VEGF: Vascular Endothelial Growth Factor
